# Combinatorial engineering of N-TIMP2 variants that selectively inhibit MMP9 and MMP14 function in the cell

**DOI:** 10.18632/oncotarget.25885

**Published:** 2018-08-10

**Authors:** Valeria Arkadash, Evette S. Radisky, Niv Papo

**Affiliations:** ^1^ Department of Biotechnology Engineering and the National Institute of Biotechnology in the Negev, Ben-Gurion University of the Negev, Beer-Sheva, Israel; ^2^ Department of Cancer Biology, Mayo Clinic Comprehensive Cancer Center, Jacksonville, Florida, USA

**Keywords:** binding specificity, directed evolution, matrix metalloproteinase, protease inhibitor, protein engineering

## Abstract

Developing selective inhibitors for proteolytic enzymes that share high sequence homology and structural similarity is important for achieving high target affinity and functional specificity. Here, we used a combination of yeast surface display and dual-color selective library screening to obtain selective inhibitors for each of the matrix metalloproteinases (MMPs) MMP14 and MMP9 by modifying the non-specific N-terminal domain of the tissue inhibitor of metalloproteinase-2 (N-TIMP2). We generated inhibitor variants with 30- to 1175-fold improved specificity to each of the proteases, respectively, relative to wild type N-TIMP2. These biochemical results accurately predicted the selectivity and specificity obtained in cell-based assays. In U87MG cells, the activation of MMP2 by MMP14 was inhibited by MMP14-selective blockers but not MMP9-specific inhibitors. Target specificity was also demonstrated in MCF-7 cells stably expressing either MMP14 or MMP9, with only the MMP14-specific inhibitors preventing the mobility of MMP14-expressing cells. Similarly, the mobility of MMP9-expressing cells was inhibited by the MMP9-specific inhibitors, yet was not altered by the MMP14-specific inhibitors. The strategy developed in this study for improving the specificity of an otherwise broad-spectrum inhibitor will likely enhance our understanding of the basis for target specificity of inhibitors to proteolytic enzymes, in general, and to MMPs, in particular. We, moreover, envision that this study could serve as a platform for the development of next-generation, target-specific therapeutic agents. Finally, our methodology can be extended to other classes of proteolytic enzymes and other important target proteins.

## INTRODUCTION

A key feature of protein–protein interactions (PPIs) with implications for both basic and applied research is the binding specificity of the interacting proteins, a property largely determined by those residues found at the interface between the two interacting polypeptides [1–3]. As the binding specificity of a protein determines its affinity to a single partner from a population of multiple targets, the ability to accurately manipulate such specificity is crucial both for understanding the mechanisms of specific PPIs and for protein engineering purposes, such as for designing specific binders and/or inhibitors of target proteins [4–7].

Methods for acquiring target specificity typically include computational approaches and mutating predicted/candidate residues and testing the resulting changes on the affinity of the protein to its specific target [8–11]. Despite considerable advances in recent years [12–15], especially the significant clinical success of target-specific therapeutic antibodies [16–19], currently available computational methods for delineating the specificity of PPIs remain lacking. As such, our ability to develop specific/selective inhibitors for clinically important proteins, for example, continues to be limited. Predicting the specificity of protein-protein interactions is more complex than predicting affinity. While specificity prediction requires both positive design (i.e., stabilization of the desired complex) and negative design (i.e., destabilization of unwanted complexes), affinity prediction considers only positive design [10, 20]. For instance, computationally saturated mutagenesis and similar classical approaches focus chiefly on single targets (namely, stabilization of the desired complex) and only allow for testing the effects of single mutations [1, 3, 9, 21, 22]. Predicting specificity by these methods is, therefore, time-consuming and laborious, as separate computations are required for all possible targets. Moreover, assessing acquired specificity calls for protein purification, sometimes of a large number of mutants [3, 9, 10], followed by binding affinity measurements for each mutant. Additional limitations of classical computational approaches for predicting specificity are inaccuracy in energy calculations due to limited sampling of possible conformational changes [23, 24] and often a failure to consider the energy of hydrogen bond formation with the solvent [12, 25]. Furthermore, mutations to proline and glycine cannot be considered by such methods, given that these mutations are likely to induce backbone conformational changes that cannot be modeled with *in silico* protocols [3]. Thus, experimentally testing the various variants that are possible so as to assess changes in specificity cannot be avoided.

With this in mind, approaches using protein-library display and selective sorting technologies that overcome some of the caveats listed above have been developed. For example, the yeast-surface display (YSD) platform, a powerful directed evolution protein engineering technology [26–31], rapidly explores all possible mutations, both single and multiple, and quantitatively screens for those binders with high target specificity [32–34]. However, in most of these methods, screening involves a fluorescently labeled target of interest in the presence of non-labeled competitor molecules [32], a scenario that could result in the selection of mutants that bind the desired target with high affinity but that also exhibit higher affinity for other targets [33]. Indeed, most currently available approaches generate high-affinity, yet not necessarily selective binders [35–37]. Moreover, in those studies that did generate selective binders, the specific inhibition of targets with high sequence and structural homology, especially within the cell, was not demonstrated.

With these considerations in mind, we have developed a dual-target selective library screen as the basis of a novel comprehensive single-step approach for identifying selective binders that strongly inhibit their targets in cells. In our strategy, two targets presenting highly similar structures and sharing a nearly identical ligand-binding epitope are fluorescently labeled using different dyes, with each target serving as a competitor for the other. In this manner, mutant binding partners that specifically interact with each target, namely variants that exhibit both high affinity to one target and low binding to the competitor target, can be identified.

In the current report, we employed our strategy to generate specificity in a non-selective matrix metalloproteinase (MMP) family inhibitor, tissue inhibitor of metalloproteinase 2 (TIMP2). TIMP2 is one of the four homologous mammalian TIMPs (TIMP1–4) that recognize the two human MMPs, MMP14 and MMP9 [38]. The inhibition of MMP proteases is of clinical value, as MMP14 and MMP9 are oncogenic [39–41]. MMP14 and MMP9 also exhibit anti-tumorigenic functions [42]. In breast carcinoma, for instance, MMP14 overexpression correlates with poor prognosis [43, 44]. Interestingly, MMP14 deficiency is lethal to mice, with MMP14 knockout mice suffering from severe abnormalities and dying shortly after birth [45, 46]. MMP9, on the other hand, was shown to promote tumor formation when expressed in stromal cells but also correlated with favorable prognosis for patients when expressed in carcinoma cells [47]. In a mouse model of breast cancer based on MCF-7 cells that do not endogenously express MMP9 and into which an adenovirus vector containing the MMP9 gene was injected, tumor regression was induced [48]. This was probably due to the ability of MMP9 to induce the anti-angiogenic endostatin expression [48, 49]. In addition, several mouse models have revealed that MMP9 deficiency increases tumor progression and invasiveness [50, 51]. At the same time, MMP14 and MMP9 fulfill additional physiologically important functions. MMP14 plays important roles in tissue regeneration and has been specifically linked with muscle renewal [52] and bone development [53]. MMP9 is important for brain development and plasticity [54]. Thus, both enzymes are involved in both pathophysiological and specific normal states, such that specific inhibition of each is crucial for therapeutics.

Like all MMP family members, MMP14 and MMP9 are multi-domain proteins that differ in domain architecture and substrate preference. However, all share a catalytic domain with a nearly identical active site containing a Zn ion. Because of the importance of MMPs in cancer, many MMP inhibitors have been designed in the past thirty years. Unfortunately, to date all have failed in clinical trials due to high toxicity [55, 56]. A major reason for the failure of these MMP inhibitors is that they were often poorly soluble and designed to bind Zn, such that they could not reach the desired target due to binding to Zn and other heavy metals in various other, unrelated proteins. In recent years, it has become clear that inhibitors with narrow or single MMP specificity hold much greater therapeutic potential than do broad specificity MMP inhibitors. Obtaining such specific inhibitors has, however, proven to be challenging.

The two MMPs can be distinguished on the basis of their distinct functional groups, with MMP9 belonging to the gelatinases and MMP14 being a membrane-type MMP. Still, the development of specific inhibitors against either protease has been challenging, probably due to the highly similar structures of MMP14 and MMP9 [57, 58]. However, as their X-ray structures are available, bioinformatics analysis of the interactions of MMP14 and MMP9 with TIMP2 is possible [59, 60]. Three-dimensional structures of TIMP-MMP complexes [61–66] have revealed that binding of TIMP to MMP mostly occurs through the ~125 amino acid-long TIMP N-terminal domain (N-TIMP) [60, 61, 63–66]. Indeed, isolated N-TIMP is a potent inhibitor of various MMPs and has been repeatedly used in place of the full-length protein in various studies [67]. Moreover, the N-TIMP (MMP-binding) interface is highly tolerant to residue substitution or the incorporation of additional amino acids [68]. As the sequence of the N-TIMP2 interface largely determines the affinity and specificity of the inhibitor to its targets [3], introducing mutations in this region would enable us to simultaneously optimize both target affinity and specificity without compromising stability, the latter being mostly governed by scaffold (non-binding interface) residues.

In the present study, examination of the N-TIMP2-MMP interface served to direct the introduction of mutations that generated N-TIMP2 variants showing improved selectivity and affinity to either MMP14 or MMP9, as evaluated in *in vitro* assays using purified proteins and in cell-based inhibition studies. We succeeded in generating an N-TIMP2 mutant library rich in affinity- and specificity-enhancing mutations. Of these, we identified the most highly selective N-TIMP2 mutants, based on their ability to inhibit either of the two proteases. We, furthermore, validated our screening results in cell-based models of MMP-dependent breast cancer cellular migration, and evaluated and compared the abilities of selected purified N-TIMP2 variants to act as functional and selective MMP inhibitors in the cell. To the best of our knowledge, this is the first report of a platform offering an effective screen of TIMPs showing specificity towards particular MMPs, with validation of the selective inhibition in cells provided.

## RESULTS

### Selective sorting of an N-TIMP2 library

**Figure 1:**
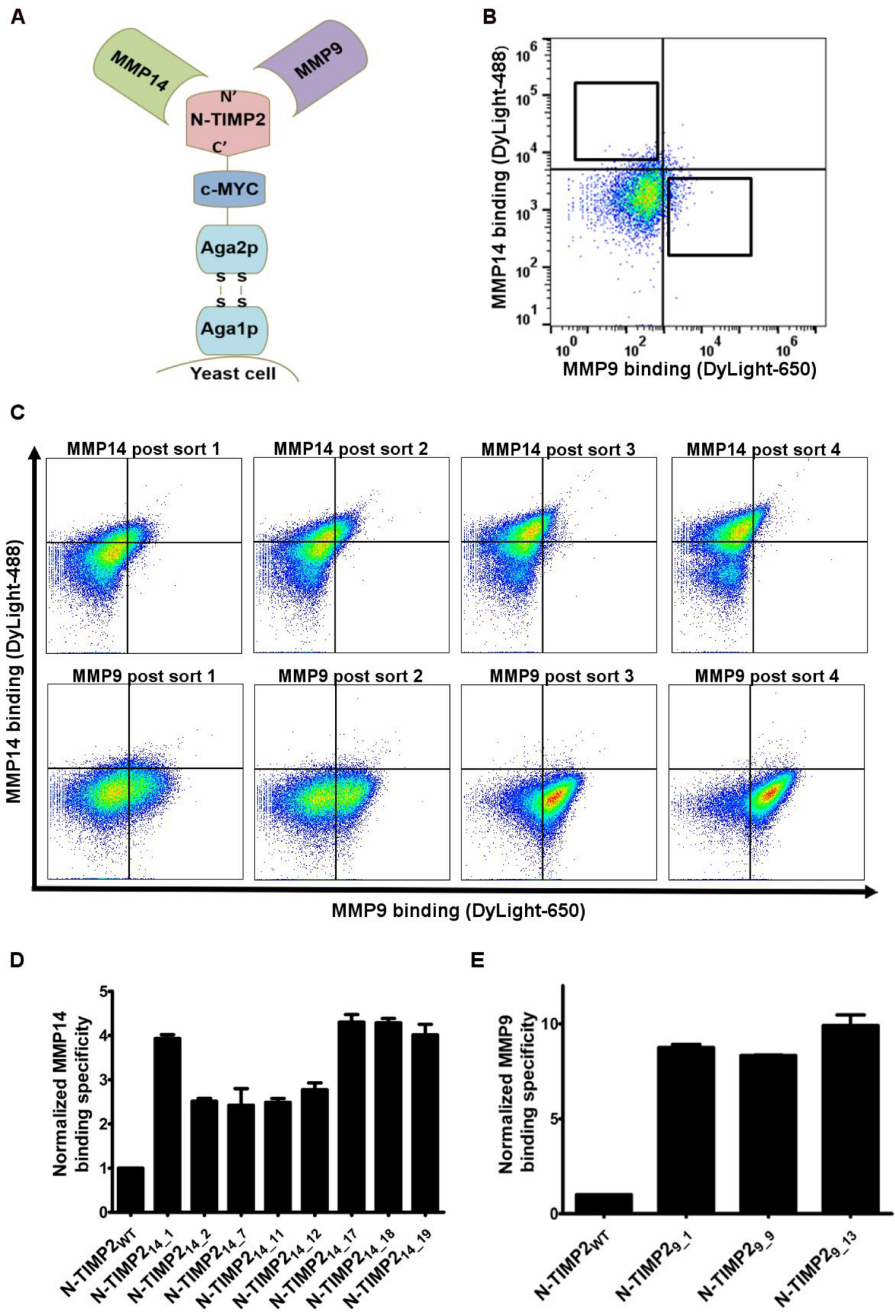
Selective library screening (**A**) Schematic representation of N-TIMP2 expressed in a yeast surface display using a pCHA construct, with MMP-14_CAT_ and MMP9_CAT_ as soluble targets. (**B**) FACS sorting of the N-TIMP2 library using 1 μM MMP14_CAT_ and 50 nM MMP9_CAT_ as targets. The y-axis shows binding to MMP14_CAT_ conjugated to DyLight488, and the x-axis shows binding to MMP9_CAT_ conjugated to DyLight-650. The squares indicate sort gates used to select the desired yeast cell populations. (**C**) Flow cytometry analysis of populations representing the four rounds of sorting using 500 nM MMP14_CAT_ and 50 nM MMP9_CAT_ as targets. (**D**) Normalized binding of individual clones with selective binding towards MMP14, identified after the fourth round of sorting. The analysis was performed using a concentration of 100 nM MMP14_CAT_ and 100 nM MMP9_CAT_. For each YSD clone, the signal for binding to MMP14_CAT_ was divided by the signal of binding to MMP9_CAT_ and the resulting ratio was normalized to the MMP14_CAT_/MMP9_CAT_ binding signal ratio of N-TIMP2_WT_. (**E**) Normalized binding of individual clones with selective binding towards MMP9, identified after the fourth round of sorting. The analysis was performed at concentration of 1 μM MMP14_CAT_ and 10 nM MMP9_CAT_. Signals for binding towards MMP9_CAT_ were divided by the corresponding signals of binding to MMP14_CAT_ and the resulting ratio was normalized to the MMP9_CAT_/MMP14_CAT_ binding signal ratio of N-TIMP2_WT_.

This study relied on the design schematically depicted in Figure 1A and 1B. Briefly, we incubated a YSD N-TIMP2 library with a mixture of MMP9 and MMP14 catalytic domains (MMP9_CAT_ and MMP14_CAT_, respectively). To evaluate the binding of members of the YSD N-TIMP2 library to either protease using fluorescence-activated cell sorting (FACS), each protease was labeled with a different fluorophore. Ideally, such sorting should reveal N-TIMP2 variants that bind exclusively to MMP14_CAT_ or MMP9_CAT_.

**Figure 2:**
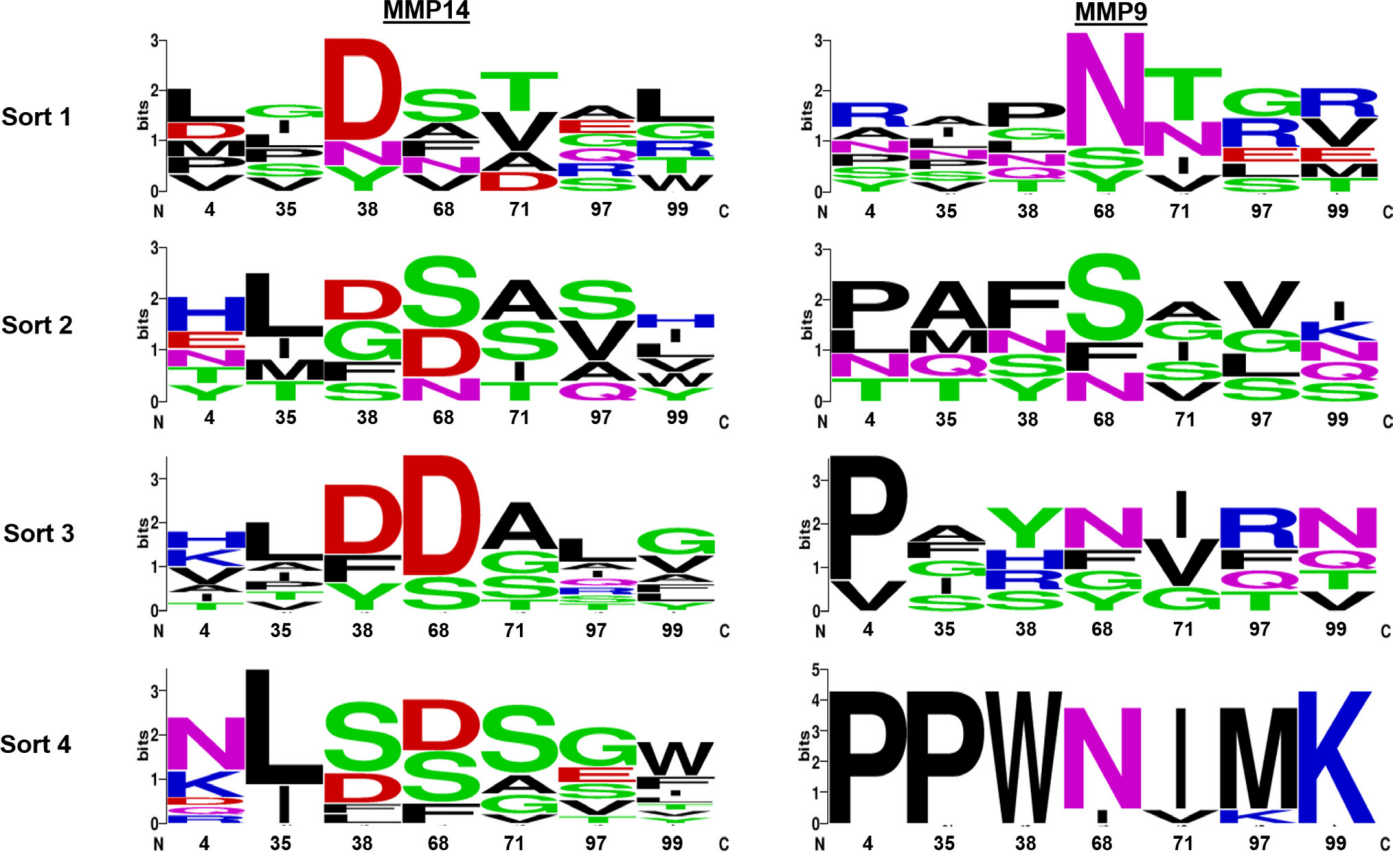
Logo summaries of the selective libraries sequenced after each selective round of sorting The height of each letter is proportional to its frequency at that position. The total height of the stack represents conservation at that position. Green, purple, blue, red and black letters, respectively, represent polar, neutral, basic, acidic and hydrophobic amino acids. The position numbers of the residues are denoted at the bottom of the figure. The logos were generated by the WebLogo server (weblogo.berkeley.edu/logo.cgi).

As a first step, a focused N-TIMP2 library involving randomization at seven positions in the binding surface of N-TIMP2 [60], found at a distance of 4 Å from MMP14 in the MMP14/TIMP2 complex [3, 60], was generated (Supplementary Figure 1). This region of TIMP2 was previously shown to well tolerate residue substitution or the incorporation of additional amino acids without compromising stability [68]. Taking this approach allowed for a reduction of the theoretical library size to ~10^8^ candidates, a size which is tractable using our yeast surface display (YSD) technique. The library was expressed on a pCHA construct that had undergone an initial round of enrichment for that fraction of clones with high expression levels, as previously described [68]. For the selective binding sorts, MMP14_CAT_ and MMP9_CAT_, each labeled with a different fluorophore (labeled and unlabeled MMPs had similar catalytic activities, Supplementary Figure 2), were simultaneously added to the yeast-displayed N-TIMP2 library (Figure 1A). In the first round of sorting, 1 *μ*M of MMP14_CAT_ conjugated to DyLight-488 was added together with 50 nM of MMP9_CAT_ conjugated to DyLight-650. Two diagonal FACS gates were used to select binders in this sort (Figure 1B), yielding the MMP14 and MMP9 high binding populations. Next, four sequential sorts were performed on each population separately. To select MMP14 high affinity clones, the concentration of MMP14_CAT_ was decreased to 250 nM in the second sort and down to 50 nM in the final sort. Likewise, the concentration of MMP9_CAT_ was increased to 100 nM in the second sort and up to 150 nM in the final round of sorting. To select for MMP9 high affinity clones, the concentration of MMP9_CAT_ was decreased to 10 nM in the second sort and down to 1 nM in the final sort, while the concentration of MMP14_CAT_ remained at 1 *μ*M. After each round of sorting, the populations were sequenced, confirming that specific mutations predominated in each population (Figure 2). In the flow cytometry analysis performed on the post-sort libraries, selective binding of each population to its designated target was confirmed (Figure 1C). After the fourth and final sort, no significant changes in binding affinity towards the targets were noted by flow cytometry analysis.

After sequencing the library obtained in the final sort, a few N-TIMP2 variants with selectivity to MMP9 and MMP14 were chosen. From the selected MMP14 high affinity binders, eight different mutants were identified, with a predominating mutation (termed N-TIMP2_14_7_) repeating in five out of twelve sequences. The individual MMP14 binding clones were assessed for selective binding to MMP14_CAT_ over MMP9_CAT_ by flow cytometry (Figure 1D), with the ratio between the signal of binding to MMP14_CAT_ and the signal of binding to MMP9_CAT_ being determined and compared with that obtained using N-TIMP2_WT_. Two clones, N-TIMP2_14_17_ and N-TIMP2_14_18_, showed the highest specific binding towards MMP14_CAT_, as compared to N-TIMP2_WT_ (~5-fold increase). As these clones showed the lowest binding towards MMP9 among clones with enhanced MMP14 affinity (Supplementary Figure 3), they were selected for purification and further examination. Of the MMP9 binders, three clones were identified, with clone N-TIMP2_9_1_ repeating in six of nine sequences (Table 1). We continued with clones N-TIMP2_9_1_ and N-TIMP2_9_13_ for production as soluble proteins, given that both showed ~10-fold higher selective binding towards MMP9, as compared to N-TIMP2_WT_ (Figure 1E).

**Table 1:** Summary of sequences of selective variants obtained after the fourth round of sorting

Clone/Position	4	35	38	68	71	97	99
N-TIMP2_WT_	S	I	N	S	V	H	T
N-TIMP2_14_1_	N	L	S	D	S	S	F
N-TIMP2_14_2_	R	I	D	D	A	T	L
N-TIMP2_14_7_	N	L	S	S	S	G	W
N-TIMP2_14_11_	K	I	D	D	G	V	V
N-TIMP2_14_12_	Q	I	D	F	G	E	I
N-TIMP2_14_17_	D	L	S	D	S	S	F
N-TIMP2_14_18_	K	L	F	F	V	E	T
N-TIMP2_14_19_	K	L	L	D	A	V	Y
N-TIMP2_9_1_	P	P	W	N	I	M	K
N-TIMP2_9_9_	P	P	W	I	I	M	K
N-TIMP2_9_13_	P	P	W	N	V	K	K

### Selective N-TIMP2 variants show improved specific *in vitro* inhibition of MMP14_CAT_ and MMP9_CAT_

**Figure 3:**
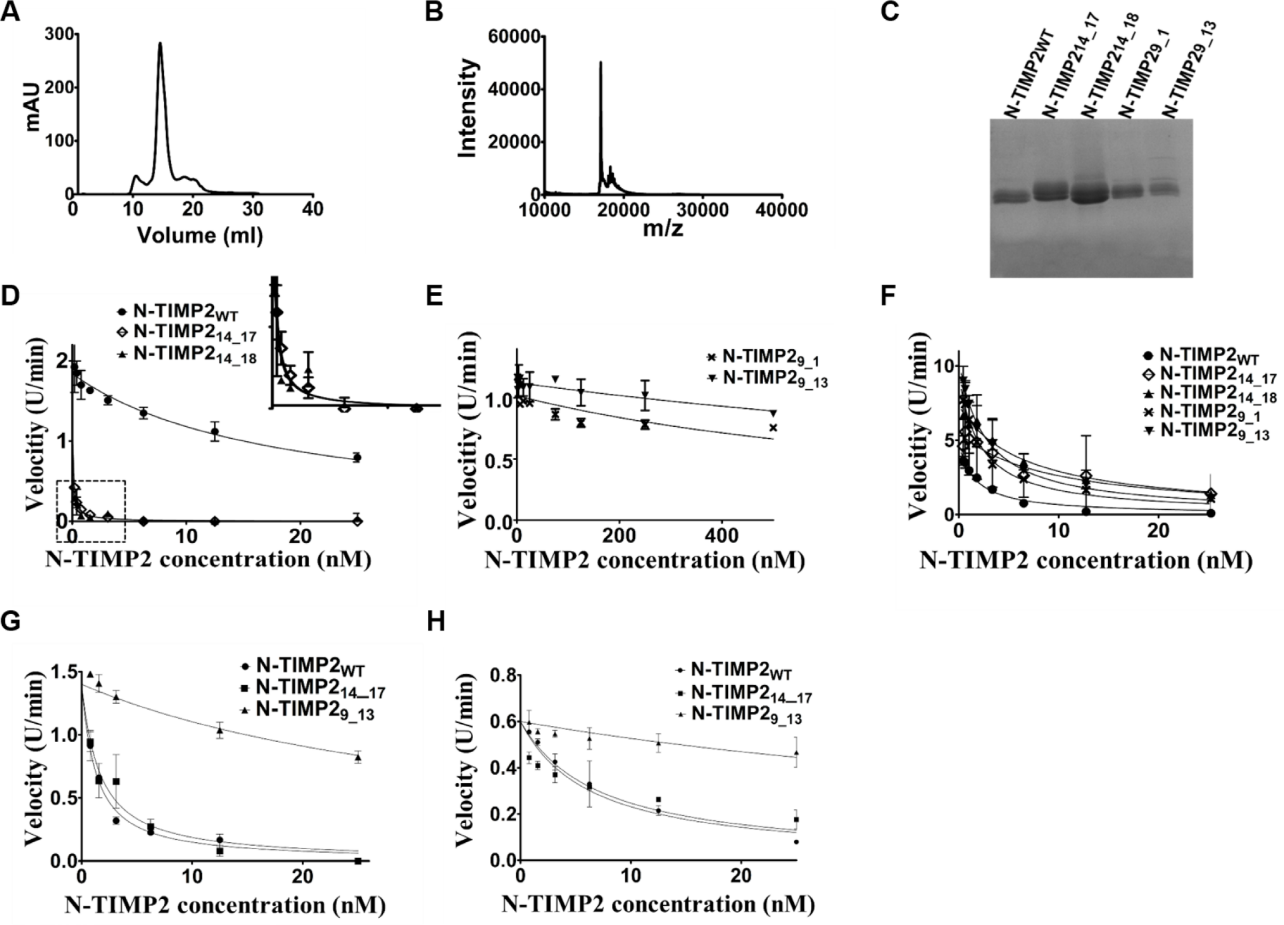
Purification of N-TIMP2 selective variants (**A**) SEC (using Superdex75) analysis of clone N-TIMP2_14_17_. (**B**) Mass spectrometry analysis of clone N-TIMP2_14_17_ after SEC. (**C**) Analysis of purified N-TIMP2_WT_, N-TIMP2_14_17_, N-TIMP2_14_18,_ N-TIMP2_9_1_ and N-TIMP2_9_13_ by 15% SDS-PAGE performed under reducing conditions. (**D**) MMP14_CAT_ inhibition by various concentrations of N-TIMP2_WT_, N-TIMP2_14_17_ and N-TMP2_14_18_. Cleavage of the fluorescent substrate was measured over time, with the velocity (slope) of the reaction as a function of inhibitor concentration being fitted to Morrison's equation to obtain the inhibition constant *Ki*. (**E**) MMP14_CAT_ inhibition by various concentrations of N-TIMP2_9_1_ and N-TIMP2_9_13_. (**F**) Inhibition of MMP9_CAT_ by N-TIMP2_WT_ and selective inhibitors. (**G**) Inhibition of MMP1_CAT_ by N-TIMP2_WT_ and the selective inhibitors. (**H**) Inhibition of MMP10_CAT_ by N-TIMP2_WT_ and the selective inhibitors.

To examine the *in vitro* inhibition of MMP14 and MMP9 by the variants, these were expressed and purified in a soluble form in the yeast *Pichia pastoris,* as previously described [68]. Briefly, the variants were expressed from the pPICZαA vector that produces versions of the proteins with a free N-terminus and C-terminal His- and c-Myc epitope tags. We purified the proteins using affinity chromatography, followed by size-exclusion chromatography (Figure 3A). The sizes and purity of the variants were confirmed by mass spectrometry and SDS-PAGE, respectively (Figure 3B, 3C). To determine the binding affinities of the purified selective N-TIMP2 variants towards MMP14 and MMP9, we performed an enzyme activity assay. Either MMP-14_CAT_ or MMP_CAT_ were incubated with increasing concentrations of N-TIMP2_WT_ (or N-TIMP2 mutants), and the cleavage of a fluorogenic substrate was determined as a function of time. The slope of each catalytic reaction was calculated and fitted to Morrison's tight binding equation (Eq. 1, see Methods) to determine the *K_i_* value (Figure 3D–3F, Table 2). N-TIMP2_WT_ inhibited MMP14_CAT_ and MMP9_CAT_ with *K_i_* values of 5 nM and 0.5 nM, respectively (Table 2), a finding consistent with previous studies [3, 68]. The two selective MMP14 inhibitors N-TIMP2_14_17_ and N-TIMP2_14_18_ inhibited MMP14 _CAT_ with *K_i_* values of 30 ± 3 pM and 24 ± 5 pM, respectively. These values correspond to affinities for MMP14 _CAT_ that were ~200-fold superior than N-TIMP2_WT_. The clones also showed 0.14- and 0.4-fold decreased affinity towards MMP9_CAT_, as compared to N-TIMP2_WT_. Thus, the calculated specificities (i.e., fold affinity enhancement), of N-TIMP2_14_17_ and N-TIMP2_14_18_ towards MMP14_CAT_ over MMP9_CAT_, relative to N-TIMP2_WT_, were ~1200 and ~500, respectively. The selective MMP9 binding variants TIMP2_9_1_ and N-TIMP2_9_13_ inhibited MMP9 with *Ki* values of 0.78 ± 0.02 nM and 1.2 ± 0.007 nM, respectively, reflecting comparable binding towards MMP9 as towards N-TIMP2_WT_. Nevertheless, these clones showed a dramatic decrease in affinity towards MMP14_CAT_, with *Ki* values of 240 ± 31 nM and 832 ± 44 nM, respectively. As a result, the calculated specificities towards MMP9_CAT_ (i.e., the ratio between the fold change of the *Ki* to MMP9_CAT_ to the fold change of the *Ki* to MMP14, relative to the same ratio obtained with N-TIMP2_WT_) of N-TIMP2_9_1_ and N-TIMP2_9_13_ were ~30 and ~70, respectively. To further evaluate target selectivity, the selective variants N-TIMP2_14_17_ and N-TIMP2_9_13_ were tested in a kinetic inhibition assay against two other MMP family members, i.e., MMP1_CAT_ (collagenase) and MMP10_CAT_ (stromelysin). The MMP14-selective variant N-TIMP2_14_17_ had the same affinity as N-TIMP2_WT_ towards MMP1 and MMP10, with *Ki* values of 0.9 ± 0.1 nM and 3.2 ± 0.46 nM, respectively (Table 3, Figure 3G, 3H). Consequently, the values of the calculated specificity for binding of TIMP2_14_17_ to MMP14_CAT_ over MMP1_CAT_ and MMP10_CAT_ were 212 and 154, respectively. On the other hand, in comparison to N-TIMP2_WT_, the MMP9-selective variant N-TIMP2_9_13_ showed a decrease in affinity towards MMP1_CAT_ and MMP10_CAT_, with *Ki* values of 18 ± 8 nM and 40 ± 14 nM, respectively (Table 3, Figure 3G, 3H). The calculated specificity of N-TIMP2_9_13_ to MMP9_CAT_ over MMP1_CAT_ and MMP10_CAT_ was, therefore, 10- and 5-fold higher, respectively.

**Table 2:** Inhibition constants (*Ki*) of MMP14 and MMP9 with N-TIMP2 selective variants

	Ki^*^ *(nM)*		Fold change of Ki^**^		
Clone	MMP14_CAT_	MMP9_CAT_	MMP14_CAT_	MMP9_CAT_	Specificity shift^***^
N-TIMP2_WT_	5 ± 1	0.5 ± 0.04			
N-TIMP2_14_17_	0.03 ± 0.003	3.58 ± 0.5	170	0.14	1175
N-TIMP2_14_18_	0.024 ± 0.005	1.25 ± 0.2	211	0.41	512
N-TIMP2_9_1_	240 ± 31	0.78 ± 0.02	0.021	0.66	31
N-TIMP2_9_13_	832 ± 44	1.2 ± 0.007	0.006	0.41	68

^*^K_i_ values (nM) of the purified variants were obtained by fitting the experimental data to Morrison's tight binding equation (Eq. 1).

^**^Fold change of *Ki* reflects the ratio between the *K_i_* of N-TIMP2_WT_ and the *K_i_* of an N-TIMP2 variant.

^***^For the MMP14-inhibiting clones N-TIMP2_14_17_ and N-TIMP2_14_18_, specificity shifts were calculated as the ratio between the fold improvement to MMP14_CAT_, as compared to MMP9_CAT_ (the specificity shift is defined as the fold change of *K_i_* for MMP14/fold change of *Ki* for MMP9). For the MMP9-inhibiting clones N-TIMP2_9_1_ and N-TIMP2_9_13_, specificity shifts were calculated as the ratio between the fold of improvement to MMP9 in comparison to MMP14 (the specificity shift is defined as the fold change of *K_i_* for MMP9/fold change of *Ki* for MMP14).

**Table 3:** Inhibition constants (*K*_*i*_) of MMP1_CAT_ and MMP10_CAT_ with N-TIMP2 selective variants

	K_i_^*^ *(nM)*		Fold change of K_i_^**^		*Specificity shift*	
Clone	MMP1_CAT_	MMP10_CAT_	MMP1_CAT_	MMP10_CAT_	MMP1_CAT_	MMP10_CAT_
N-TIMP2_WT_	0.7 ± 0.1	3.5 ± 0.8				
N-TIMP2_14_17_	0.92 ± 0.15	3.2 ± 0.4	0.8	1.1	212^#^	154^#^
N-TIMP2_9_13_	18 ± 8	40 ± 14	0.04	0.08	10^+^	5^+^

^*^K_i_ values (nM) of the purified variants were obtained by fitting the experimental data to Morrison's tight binding equation (Eq. 1).

^**^Fold change of *Ki* reflects the ratio between the *K_i_* of N-TIMP2_WT_ and the *K_i_* of an N-TIMP2 variant.

^#^ For the MMP14-inhibiting clone N-TIMP2_14_17_, specificity shifts were calculated as the ratio between the fold improvement to MMP14_CAT_, as compared to MMP1_CAT_ and MMP10_CAT_ (the specificity shift is defined as the fold change of *K_i_* for MMP14/fold change of *Ki* for MMPX, where X designates either MMP1_CAT_ or MMP10_CAT_).

^+^ For the MMP9-inhibiting clone N-TIMP2_9_13_, specificity shifts were calculated as the ratio between the fold of improvement to MMP9_CAT_ in comparison to MMP1_CAT_ and MMP10_CAT_ (the specificity shift is defined as the fold change of *K_i_* for MMP9/fold change of *Ki* for MMPX, where X designates either MMP1_CAT_ or MMP10_CAT_).

### N-TIMP2 variants selective to MMP14 inhibit MMP2 activation in U87MG cancer cells

**Figure 4:**
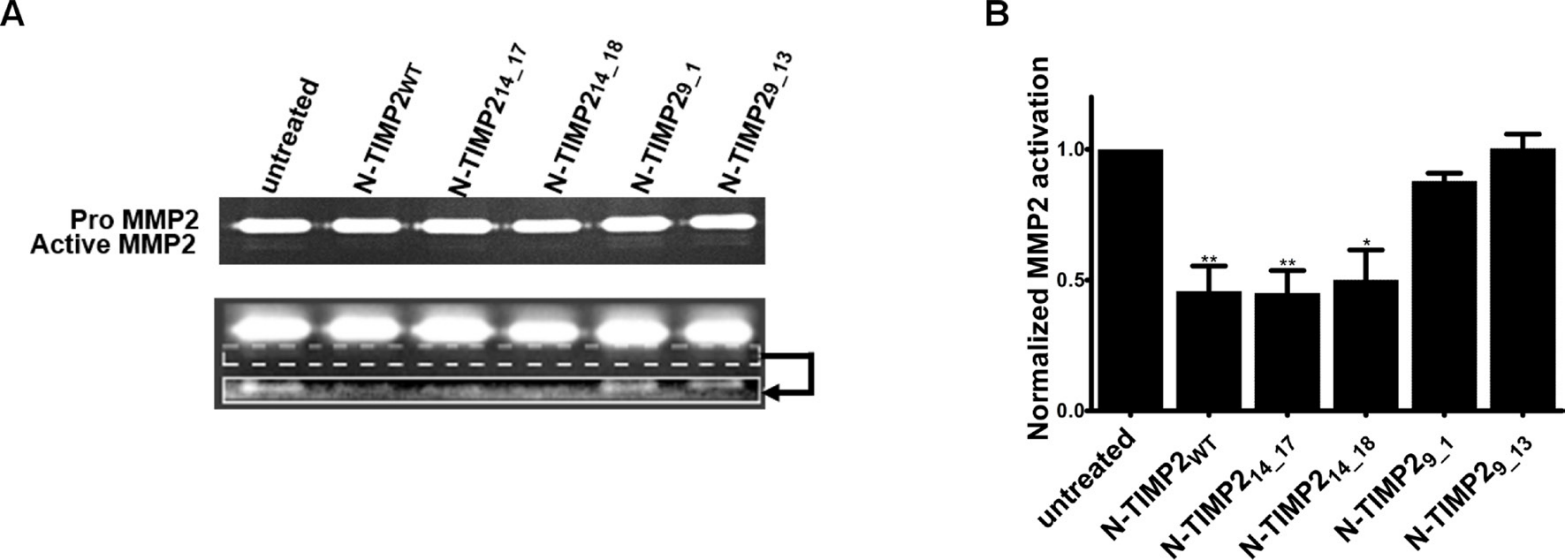
Inhibition of MMP2 activation by MMP14 (**A**) Representative results of gelatin zymography analysis of media collected from U87MG cells incubated with 100 nM of the inhibitors for 48 h and resolved by SDS-PAGE. The bands in the top panel reflect inactive pro-MMP2, while the boxed region in the bottom panel contains bands that reflect active MMP2. The bottom panel in fact corresponds to the same gel used to generate the top panel, albeit after enhanced exposure and with increased sensitivity of detection. Specifically, to generate the lower panel, only the upper bright bands in gel were covered and a picture of the lower bands with 6-fold increased exposure and a 2-fold increase in sensitivity was taken. In addition, the contrast was increased. (**B**) Quantification of band intensity from the gel containing active MMP2, normalized to the intensities of bands from gels of control (untreated) cells. The experiment was repeated three times; means and standard error are given. ^*^P < 0.05, ^**^P < 0.01, as determined by *t* tests comparing the indicated condition versus the untreated control.

MMP2 is processed to its active form upon cleavage by MMP14. Active MMP2 in turn promotes invasion by and metastasis of different cancers [69, 70]. Accordingly, inhibition of MMP14 was previously shown to inhibit the activation of MMP2 [71]. To examine whether our selective clones could selectively inhibit the activity of MMP14 in a cancer cell model, we performed a gelatin zymography assay with U87-malignant glioma (U87MG) cells, which naturally express high levels of MMP14, MMP9 and MMP2 [70, 72, 73]. U87MG cells were incubated in serum-free medium for 48 h in the presence or absence of the inhibitors. Thereafter, the media were collected and resolved by SDS-PAGE with 1% gelatin embedded in the gel, allowing for the inactive and active forms of MMP2 to be visualized (Figure 4A). N-TIMP2_WT_ and the MMP14-selective inhibitors successfully reduced the amounts of active MMP2 (Figure 4), with 45% inhibition being seen with N-TIMP2_WT_ and N-TIMP2_14_17_, and 50% inhibition being obtained with N-TIMP2_14_18_. The selective MMP9-inhibiting variant N-TIMP2_9_13_ showed no inhibition of MMP14-induced MMP2 activation, consistent with its low affinity to MMP14.

### Selective MMP9 inhibitors inhibit the gelatinolytic activity of MMP9

**Figure 5:**
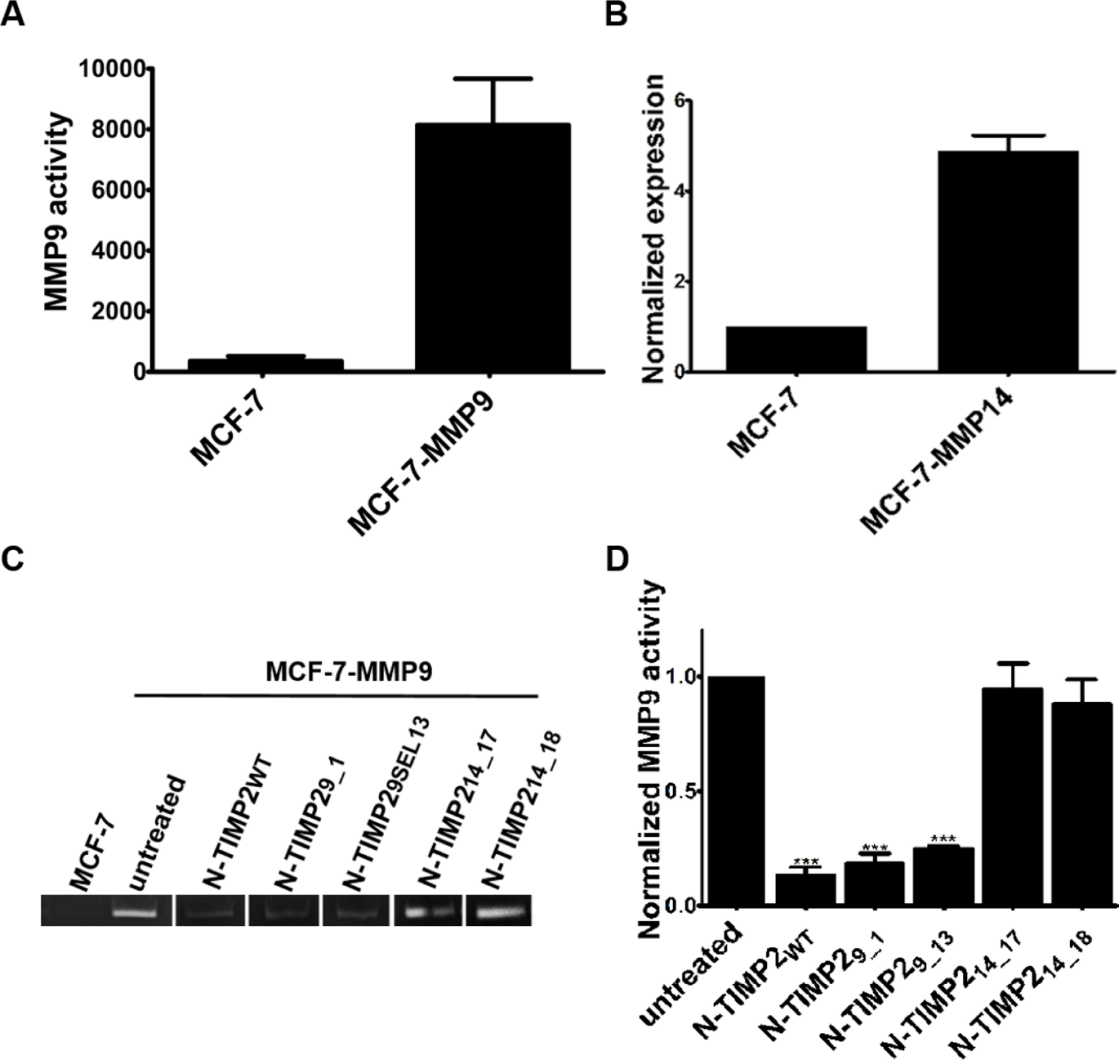
Gelatin degradation by MMP9 produced in MCF-7-MMP9 cells (**A**) Band intensity quantification of gelatin zymography analysis of media collected from MCF-7 and MCF-7-MMP9 cells. Band intensities were quantified by ImageJ software. (**B**) Normalized expression of MMP14 in MCF-7 and MCF-7-MMP14 cells was confirmed by flow cytometry using rabbit anti-MMP14 antibodies and secondary goat anti-rabbit PE antibodies. (**C**) Representative results of gelatin zymography analysis of media collected from untransfected MCF-7-WT cells and MCF-7-MMP9 cells stably expressing MMP9 resolved in SDS-PAGE and treated with 100 nM of the inhibitors. (**D**) Quantification of band intensity normalized to the intensity obtained with control (untreated) cells. All the the experiments were performed in triplicate; means and standard error are shown. In D, ^***^P < 0.001, as determined in a *t* test comparing activity in the presence of the various inhibitors versus the untreated control.

Many cancer cell lines express both MMP14 and MMP9 [74, 75]. To assess the specific effects of our selective inhibitors on each MMP, we utilized the MCF-7 breast cancer cell line that is naturally MMP14- and MMP9-deficient [76, 77]. First, we stably transfected the cells with a full-length MMP9 construct to generate MCF-7-MMP9 cells. Since MMP9 is a secreted gelatinase, we confirmed its expression by gelatin zymograpy (Figure 5A, 5C). As expected, wild type MCF-7 cells that did not express MMP9 showed no such activity, whereas MCF-7-MMP9 cells exhibited gelatin degradation (Figure 5A, 5C, 5D). To address the selective inhibition of gelatin degradation by our N-TIMP2-specific MMP14 and MMP9 inhibitors, the supernatant of MCF-7-MMP9 cells was resolved by SDS-PAGE using gelatin-embedded gels and treated with 100 nM of the inhibitors. The strongest inhibition of gelatin-degrading activity (~86%) was obtained upon treatment with N-TIMP2_WT_ (Figure 4B). The MMP9-selective N-TIMP2_9_1_ and N-TIMP2_9_13_ proteins caused ~82% and ~75% inhibition of gelatin degradation activity, respectively. This finding is consistent with the slightly higher affinity of N-TIMP2_WT_ towards MMP9, as compared to the affinities of the two clones. Also as expected, the MMP14-binding clones did not cause inhibition of MMP9 gelatinolytic activity.

### Selective inhibition of MMP14- and MMP9-induced MCF-7 cell migration

**Figure 6:**
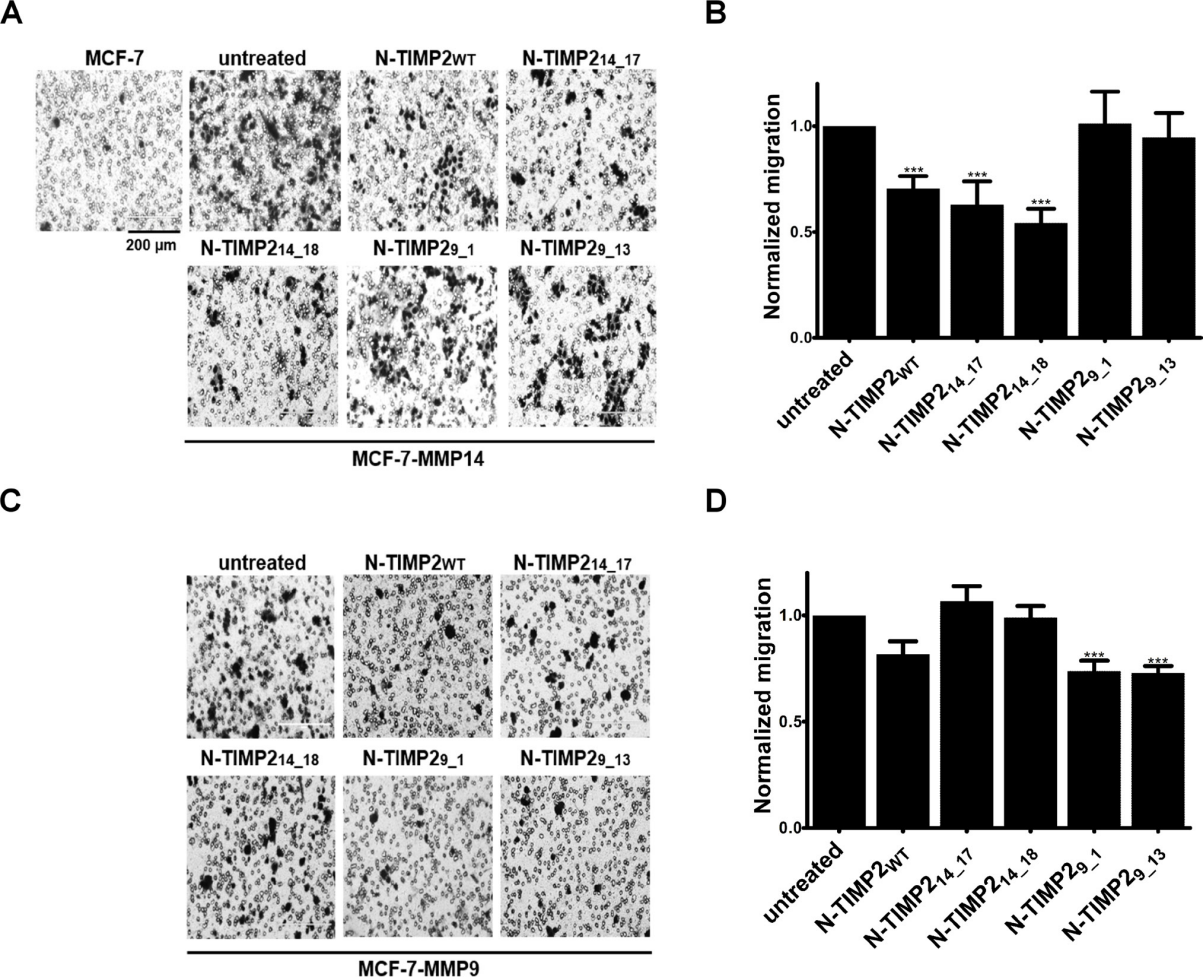
Selective inhibition of MMP14- and MMP9-induced migration (**A**) Representative micrographs of migrating MCF-7 and MCF-7-MMP14 cells in the presence or absence of 100 nM of the N-TIMP2 inhibitors. The cells were stained with Dipp Kwik Differential Stain and visualized by light microscopy using a ×20 magnification lens. (**B**) Calculated fold of migration of MCF-7-MMP14 cells. The cells were counted using ImageJ software and normalized to counts of untreated cells. (**C**) Representative micrographs of migrating MCF-7-MMP9 cells in the presence or absence of 100 nM of the N-TIMP2 inhibitors. The cells were visualized as in A. (**D**) Calculated fold of migration of MCF-7-MMP9 cells. The cells were counted as in B. The experiment was repeated three times; means and standard error are shown. ^***^P < 0.001, as determined by a *t* test comparing inhibition in the presence of the various inhibitors versus the untreated control.

Wild type MCF-7 cells lack migratory abilities [78] due to a lack of MMP14 expression [77]. At the same time, stable expression of MMP14 in these cells induced migration and invasion [77]. Previous works also showed that induction of MMP9 expression increased the invasiveness of MCF-7 cells [79]. We performed trans-well migration assays for the purpose of exploring the effects of our selective inhibitors on the migration of MCF-7 cells stably expressing either MMP14 (Figure 5B) or MMP9. In the assay, wild type MCF-7 cells did not migrate through the membrane. In contrast, significant migration was observed with MCF-7-MMP14 and MCF-7-MMP9 cells (Figure 6A–6C). Our N-TIMP2 variants could specifically inhibit the migration induced in MCF-7-MMP14 and MCF-7-MMP9 cells by MMP14 or MMP9, respectively, in a dose-dependent manner (Figure 6 and Supplementary Figure 4). In the MMP14-expressing cells, N-TIMP2_WT_ and N-TIMP2_14_17_ inhibited migration by 30% and 38%, respectively, while N-TIMP2_14_18_ reduced migration by ~50%. As expected, both MMP9-binding variants (N-TIMP2_9_1_ and N-TIMP2_9_13_) did not inhibit MCF-7-MMP14 cell migration. The same trend was observed with MCF-7-MMP9 cells. The MMP14-binding variants did not inhibit the migration of MCF-7-MMP9 cells, whereas treatment with the MMP9-selective inhibitors N-TIMP2_9_1_ and N-TIMP2_9_13_ caused ~30% inhibition (Figure 6D).

## DISCUSSION

In this report, we described a new strategy for generating binding specificity in protein ligands through a combination of YSD and selective library screening against a target pair, each labeled with a different fluorophore. Employing the TIMP2/MMP system as the PPI of interest, we demonstrated how our strategy can be used to develop selective inhibitors for proteins that share high sequence homology and structural similarity in a rapid, single-step and cost-effective manner. Several studies in which selective binders were obtained using different library screening and affinity maturation approaches have been described for both basic and applicative research. These include the development of the neurotensin receptor, a GPCR family member, to bind its neurotensin (NT) agonist but not the small molecule SR 48692 antagonist [80], Fc-conjugated cytotoxic T lymphocyte–associated protein 4 (CTLA4-Ig) engineered to selectively bind either CD86 [81] or CD80 [82], and cystine knot peptides with selective binding towards specific types of integrins [34, 83]. These approaches, however, employed screens either against single targets or against a labeled desired target in the presence of other unlabeled undesired targets [33, 34, 83]. As such, these methods usually produced mutants that bind the desired target with high affinity yet which also exhibit higher affinity for other off targets [33, 81, 82]. For example, affinity maturation of the human Agouti-related protein (AgRP) against α_v_β_3_ integrin also resulted in high affinity to α_v_β_5_, and α_5_β_1_ integrins [84]. Nevertheless, in those cases that yielded selective binders, as in the follow-up study where the same AgRP was evolved to specifically bind α_IIb_β_3_ integrin over αvβ_3_, αvβ_5_ and α_5_β_1_ [34], neither specificity switching variants were identified nor was major biological characterization validating their specific binding provided [83, 85]. In the present study using our novel strategy, we developed specificity-switching variants of N-TIMP2 that function as selective MMP9 and MMP14 inhibitors, with the selective activity being confirmed by *in vitro* inhibition, cell-based gelatin zymography and cell-based migration experiments in cells that simultaneously express MMP9 and MMP14 or only one of these targets.

The MMP family represent an ideal group of targets to demonstrate our strategy because inhibiting MMPs is of clinical value, as both MMP9 and MMP14 are involved in cancer progression [41, 86]. Moreover, MMP9 is also involved in angiogenesis inhibition [49, 87], while MMP14 is involved in muscle [52] and bone development [88]. We and others have previously used computational methods [3, 68, 89, 90] and X-ray crystallography [60, 66, 89] to explore the structures of these proteases, in some cases identifying distinguishing features of individual MMPs that could be exploited for developing highly selective inhibitors [3, 60, 68, 91]. Prior to the present, however, inhibitors capable of discriminating between MMPs had yet to be described.

N-TIMP2 attracted our interest as a scaffold for engineering selective MMP inhibitors due to the marked sequence diversity among TIMP family members (only 40–50% sequence identity), and given that all possess canonical binding interfaces that are highly tolerant to residue substitution or the incorporation of additional amino acids [3, 68, 92]. Because the sequences of the canonical binding interface largely determine the affinity and specificity of an inhibitor to its target [65, 93], exploiting N-TIMP2 as a scaffold offers a unique opportunity to optimize target affinity and selectivity without compromising stability [94]. In addition, the affinity of wild-type N-TIMP-2 (N-TIMP2_WT_) to MMP9_CAT_ and MMP14_CAT_, is comparable (*K*_i_ (M) = (0.5 ± 0.04) × 10^−9^, and (5 ± 1) × 10^−9^ for MMP9_CAT_ and MMP14 _CAT_, respectively [3, 68]). These features make N-TIMP2_WT_ an optimal model scaffold for engineering binding specificity. The lack of large differences in terms of specificity toward MMP9 and MMP14 is a good starting point for changing/shifting relative specificity. At the same time, the slightly lower specificity of N-TIMP2 toward MMP14 makes it a good target for engineering and testing specificity switches.

As validation of the utility of our approach, we showed that the YSD affinities of the selected N-TIMP2 clones of MMP9_CAT_ and MMP14 _CAT_ correlated well with the target-binding specificity of the purified protein variants in solution, as measured by competitive inhibition studies. For example, the YSD binding analysis predicted a 10-fold specificity enhancement of N-TIMP2_9_1_ binding MMP9_CAT_ over MMP14_CAT_, as compared with N-TIMP2_WT_, which was in qualitative agreement with the 30-fold enhancement in the *K*_i_ values measured for the soluble proteins. Similar agreement between YSD binding analysis results and measured *K*_i_ values was reported for the mesotrypsin-binding triple mutant amyloid β-protein precursor inhibitor (APPI) recently developed in our group, which showed a greater affinity increase in the soluble form than in YSD experiments [95].

An important and novel finding reported here was that in our system, mutation-tolerant positions complied with the cold-spot definition, albeit for specificity (i.e., selective binding to a specific MMP) rather than for affinity (i.e., increased binding to that MMP). As shown by our YSD binding assays, all of the mutants that were sorted for selective MMP9_CAT_ binding did not exhibit improved affinity to MMP9_CAT_. Nonetheless, all showed improved MMP9 specificity (or MMP14 specificity, in the case of mutants sorted for selective MMP14 binding). Overall, ~5–10-fold improved specificity, either for MMP9 over MMP14, were noted in our analysis for the YSD selective clones. These results are likely directly derived from our specificity maturation approach.

We also validated the specificity changes attained using selected purified soluble N-TIMP2 variants and *in vitro* and cell-based MMP inhibition experiments. We generated and purified soluble versions of the N-TIMP2 variants that conferred an improvement in specificity in terms of binding to MMP9_CAT_, as opposed to MMP14_CAT_. These variants include those mutants for which the YSD binding analysis predicted specificity improvement from one MMP to the other. In comparison to N-TIMP2_WT_ that showed 10-fold difference in binding to MMP9_CAT_ vs MMP14_CAT_, the best mutant (namely, N-TIMP2_9_13_) assessed in our *in vitro* inhibition experiments exhibited ~700-fold better affinity in favor of MMP9_CAT_ over MMP14_CAT_. In addition, the improvement in affinity of the N-TIMP2_14_17_ mutant to MMP14 over MMP9 corresponded to 120- times enhanced affinity, reflecting a specificity switch from MMP9 to MMP14 that was also predicted in the YSD binding analysis.

In addition to the improved binding specificity of our selective mutants towards MMP9_CAT_ (or MMP14_CAT_) relative to the other protease when studied as purified proteins, biological activities of the inhibitors in cells also correlated to the binding specificity results. When tested for their ability to inhibit the gelatinolytic activity of MMP9, our MMP9-selective N-TIMP2 variants showed selective inhibition of MMP9 stably expressed by MCF-7-MMP9 cells. The same effect was observed in a different experimental system in which cell migration/mobility, previously shown to be dependent on the expression of MMP9 and MMP14, of MCF-7 cells stably expressing MMP9 was measured [77, 79]. Inhibited mobility was caused by N-TIMP2_WT_ and the specific MMP9-inhibiting N-TIMP2 protein variants at 100 nM concentrations, whereas no inhibition was observed with any of the MMP14-specific N-TIMP2 variants. The same selectivity in inhibition was also observed in mobility assays of MMP14-expressing cells, where the specific MMP14 inhibitors inhibited the MCF-7-MMP14 cell mobility, with clone N-TIMP2_14_18_ causing the highest degree of inhibition, and with none of MMP9-selective N-TIMP2 variants inhibiting this mobility. Moreover, in U87-MG cells, MMP2 activation was inhibited by the selective MMP14 inhibitors but not by the MMP9 inhibitors, suggesting that only the MMP14 inhibitors bound to MMP14, the activity of which is needed for MMP2 activation.

Finally, analysis of the libraries after sorting was limited by the number of sequences that could be obtained from single colonies. Nonetheless, we judged it to be unnecessary to sequence additional clones, because the library size had decreased significantly by the fourth round of selection in the case of the selective/competitive screens. We felt that further sequencing would not have identified greater mutational diversity in the final sorting stages. Despite the relatively small number of sequence clones, the use of a focused library and degenerate codons at multiple mutation-tolerant positions in the N-TIMP2 ligand allowed for the incorporation of multiple mutations at these positions and enabled us to identify a combination of mutations in the N-TIMP2 sequence that improved the binding specificity of this ligand towards its targets. In addition, the use of a focused library covered a large portion of the theoretical mutational diversity and provided a comprehensive screen of the binding interface. However, generating the library by using a combination of site-specific saturation mutagenesis on the canonical N-TIMP2 interface, together with random mutations at other positions in the gene (using error-prone PCR, for example) could allow us in future to identify residues that are distant from the interaction site. These specificity-improving mutations may also be beneficial for designing targeted therapeutics for different types of cancer or other diseases, as they can potentially specifically inhibit a particular MMP so as to minimize toxic effects.

In summary, we have established a methodology integrating a combination of focused combinatorial methods for library design and an YSD technique for dual-labeled target selective library screening under competitive conditions and employed this approach for the design of selective MMP inhibitors. In future, this methodology can be applied to the design of other protease inhibitors with stronger affinity or greater functionality. In more general terms, this work offers a model for future design projects in which data regarding the contributions of single positions on binding affinity is available. Such positions can be mutated and successfully combined in a combinatorial manner, as shown here, with the use of the YSD setup to obtain mutants possessing additional desirable characteristics.

## MATERIALS AND METHODS

### Selective screening of an N-TIMP2 focused library

A focused N-TIMP2 library with random mutations at seven positions of the N-TIMP2 gene affecting residues in the binding interface (4, 35, 38, 68, 71, 97, 99) (PDB:1BUV) [60] was purchased from GenScript (Piscataway, NJ). Briefly, the library was prepared using NNS (where N represents A, C, T or G nucleotides, and S represents C and G) degenerate codons that were used to mutate the above mentioned positions in the N-TIMP2_WT_ gene. The library was expressed in the yeast surface display (YSD) system [96] using the pCHA vector introduced into *Saccharomyces cerevisiae* EBY100, as previously described, followed by expression enrichment [68]. A library size of 8×10^6^ clones was confirmed by performing serial dilutions on selective SDCAA plates (2% dextrose, 0.67% yeast nitrogen base, 0.5% bactocasamino acids, 1.47% sodium citrate, 0.429% citric acid monohydrate, pH 4.5, 1.5% agar). For sorting, the yeast libraries were grown in expression-inducing SGCAA medium (2% galactose, 0.67% yeast nitrogen base, 0.5% Bacto casamino acids, 0.54% Na_2_HPO_4_·H_2_O, 0.86% NaH_2_PO_4_) at 30° C overnight. The cells, numbering ten times the library size (or 10^6^ at least), were collected and washed with a buffer containing 50 mM Tris-HCl, pH 7.5, 100 mM NaCl, 5 mM CaCl_2_ and 1% bovine serum albumin. In the first selective sort, the library was incubated with both 1 μM MMP14_CAT_ conjugated to DyLight-488 and 50 nM MMP9_CAT_ conjugated to DyLight 650 for 1 h at room temperature. The selective screen was performed on an iCyt Synergy FACS apparatus (Sony Biotechnology, San Jose, CA), and about 1% of the bound populations were selected using polygonal gates. For subsequent screens, the following combinations of simultaneously applied MMP14_CAT_ and MMP9_CAT_ were used: for the selection of MMP14_CAT_-binders, 250 nM MMP14 _CAT_ and 100 nM MMP9_CAT_ were used in the second sort, and 50 nM MMP14_CAT_ and 150 nM MMP9_CAT_ were used in the third and the fourth and final sort. For the selection of MMP9-binders, 1 μM MMP14_CAT_ and 10 nM MMP9_CAT_ were used in the second sort, while in the third and fourth screens, concentrations of 1 μM and 1 nM MMP9_CAT_ were applied, respectively. For flow cytometry analysis, the same labeling protocol as for the flow cytometry sorting was applied, using 10^6^ cells per analysis. The target concentrations used were 100 nM MMP14_CAT_ and 100 nM MMP9_CAT_ for the MMP14-specific clones and 1 μM MMP14_CAT_ and 10 nM MMP9_CAT_ for the MMP9-specific clones. Flow cytometry analysis was performed on an Accuri C6 flow cytometer (BD Biosciences, San Jose, CA).

### DNA sequencing

For sequencing the N-TIMP2 selective libraries after each round of sorting, the plasmid DNA was purified using a Zymoprep yeast plasmid miniprep I kit (ZymoResearch, Irvine, CA). The plasmid was then introduced into electro-competent *Escherichia coli* cells which were grown on LB-agar plates containing ampicillin (100 *μ*g/ml). Thereafter, about 20 colonies were transferred to liquid LB culture medium containing ampicillin and grown overnight at 37° C. The plasmid was purified using a HiYield plasmid mini kit (RBC Bioscience, Taiwan) according to the manufacturer's protocol. The plasmids were sequenced by the Sanger sequencing method (Genetics Unit, NIBN), and the sequences were analyzed using Geneious R7 software (Biomatters, Auckland, New Zealand).

### Protein purification

The portion of the human gene encoding the MMP14 catalytic domain (MMP14_CAT_, residues 112–292) was introduced into the pET3a vector that adds a C-terminal 6×His tag and used to transform *E. coli* BL-21 (DE3) cells. Tagged MMP14_CAT_ was purified as previously described [65, 97]. The human MMP9 catalytic domain (MMP9_CAT_) lacking the fibronectin-like domain (residues 107–215 and 391–443) was a generous gift from Irit Sagi (Weizmann Institute of Science, Rehovot, Israel). Purified MMP14_CAT_ and MMP9_CAT_ were labeled with DyLight-488 (Thermo Fisher, Waltham, MA) and DyLight-650 (Thermo Fisher), respectively, as previously described [68]. The catalytic activities of the labeled MMP14_CAT_ and MMP9_CAT_ enzymes were confirmed in an assay performed in TCNB buffer (50 mM Tris-HCl, pH 7.5, 100 mM NaCl, 5 mM CaCl_2_, and 0.05% Brij) with a final concentration of 15 *μ*M of the fluorogenic substrate Mca-Pro-Leu-Gly-Leu-Dpa-Ala-Arg-NH_2_TFA, where Mca is (7-methoxycoumarin-4-yl)acetyl, Dpa is N-3-(2,4-dinitrophenyl)-L-2,3-diaminopropionyl and TFA is trifluoroacetic acid (Merck Millipore, Burlington, MA,). Fluorescence was monitored for 1 h using a Synergy 2 plate reader with 340/30 excitation and 400/30 emission filters (BioTek, Winooski, VT) at 37° C. The catalytic domains of MMP1 and MMP10 (MMP1_CAT_ and MMP10_CAT_, respectively) were purified as previously described [66, 91].

N-TIMP2_WT_ and the selective variants were produced in the *P. pastoris* X33 strain. The genes were first amplified with forward primers for N-TIMP2_WT_ (5’-GGTATCTCTCGAGAAAAGATGCAGCTGCT CCCCG-3’) and for the N-TIMP2_14_17_, N-TIMP2_14_18_, N-TIMP2_9_1_ and N-TIMP2_9_13_ variants (5’- GGGTATCTCTCGAGAAAAGATGCAGCTGCGAC-3’, 5’- GGGTATCTCTCGAGAAAAGATGCAGCTGCAA G-3’, 5’- GGGTATCTCT CGAGAAAAGATGCAGCTGC CCC -3’ respectively), and a common reverse primer (5’-GCTGGCGGCCGCCT CGCAGCCCATCTGGTA-3’). The amplified N-TIMP2 variants were digested with XhoI and NotI restriction enzymes (New England Biolabs, Ipswich, MA), as was the pPICZαA vector that contains a zeocin resistance gene, the AOX1 promoter at the 5’-end of the insert and which introduces a 6×His tag at C-terminus of the translated protein. The inserts and vector were ligated and the resulting plasmid was introduced into electro-competent *E. coli* cells. The transformed bacteria were plated on LB agar plates containing 50 μg/ml zeocin (Invitrogen, Grand Island, NY). The sequences of extracted plasmids from a few clones per each N-TIMP2 mutant were verified (Genetics Unit, NIBN). Then, 100 *μ*g of plasmid containing the correct sequence were linearized upon digestion with the SacI restriction enzyme (New England Biolabs). Plasmids containing the N-TIMP2 variants were transformed into electro-competent *P. pastoris* X33 according to the pPICZα protocol (Invitrogen). The transformed yeast were grown on YPDS plates (2% peptone, 1% yeast extract, 2% D-glucose, 1 M sorbitol, 2% agar) for 72 h at 30° C. For expression, several colonies encoding each of the N-TIMP2 variants were grown in 5 ml liquid BMGY medium (2% peptone, 1% yeast extract, 0.23% K_2_H(PO_4_), 1.1812% KH_2_(PO_4_), 1.34% yeast nitrogen base, 4 × 10^–5^% biotin, 1% glycerol). After overnight growth at 30° C, the cultures were grown in inductive BMMY medium (2% peptone, 1% yeast extract, 0.23% K_2_H(PO_4_), 1.1812% KH_2_ (PO_4_), 1.34% yeast nitrogen base, 4×10^–5^% biotin, 0.5% methanol) for 72 h at 30° C, with daily addition of 1% methanol. Over-expression of the secreted proteins was determined by western blot, using a 1:3000 dilution of mouse anti-6×His primary antibodies (Abcam, Cambridge, UK), followed by a 1:5000 dilution of anti-mouse secondary antibodies conjugated to alkaline phosphatase (Jackson ImmunoResearch, West Grove, PA), and detection by incubation in 2 ml of 5-bromo-4-chloro-3-indolyl phosphate reagent (Sigma-Aldrich, St. Louis, MO). Large-scale production of the proteins was performed by growth of the N-TIMP2-expressing yeast clone exhibiting the highest amount of the desired protein for 72 h in BMMY medium, with 1% methanol added daily. During purification of the proteins, the yeast cell suspensions were centrifuged at 3800g for 10 min and the supernatant was filtered, followed by addition of 500 mM NaCl and 10 mM imidazole, pH 8.0. The supernatants were incubated for 1 h at 4° C and loaded onto nickel-nitrilotriacetic acid-Sepharose beads (Invitrogen), washed with 50 mM Tris-HCl, pH 7.5, 100 mM NaCl, and 10 mM imidazole, eluted with 20 ml of 50 mM Tris-HCl, pH 7.5, 100 mM NaCl, 300 mM imidazole, and 5 mM CaCl_2_, and concentrated using a Vivaspin centrifugal concentrator with a 3-kDa cutoff (GE Healthcare Life Sciences, Pittsburgh, PA). The proteins were further purified using a Superdex 75 column with elution buffer (50 mM Tris-HCl, pH 7.5, 100 mM NaCl and 5 mM CaCl_2_) in an ÄKTA pure instrument (GE Healthcare Life Sciences) and separated by 15% SDS-PAGE under reducing conditions. Protein bands were visualized by staining with Instant Blue (CBS Scientific, Del Mar, CA). Protein samples were concentrated using a Vivaspin centrifugal concentrator with a 5-kDa cutoff (GE Healthcare Life Sciences) and subjected to mass spectrometry analysis (Ilse Katz Institute for Nanoscale Science and Technology, Ben-Gurion University). Protein concentrations were determined by UV-Vis absorbance at 280 nm, using a NanoDrop Spectrophotometer (Thermo Scientific), with an extinction coefficient (ε_280_) of 13,500 M^-1^cm^-1^ for N-TIMP2_WT_ and its variants. An average concentration of about 1 mg/L protein for all variants was measured.

### MMP inhibition studies

N-TIMP2_WT_ and its variants were tested for inhibitory activity against 0.0075 nM MMP-14_CAT_ and 0.0075 nM MMP-9_CAT_. For MMP14_CAT_ inhibition, 0.4–25 nM of N-TIMP2_WT_, N-TIMP2_14_17_, N-TIMP2_14_18_ and 1.5–500 nM of N-TIMP2_9_1_ and N-TIMP2_9_13_ were used. For inhibiting MMP9_CAT,_ MMP1_CAT_ and MMP10_CAT_, 0.4–25 nM of the inhibitors were used. The inhibition assays were performed in TCNB buffer (50 mM Tris-HCl, pH 7.5, 100 mM NaCl, 5 mM CaCl_2_, and 0.05% Brij) for 1 h at 37° C. Thereafter, the fluorogenic substrate Mca-Pro-Leu-Gly-Leu-Dpa-Ala-Arg-NH_2_·TFA, where Mca is (7-methoxycoumarin-4-yl)acetyl, Dpa is N-3-(2,4-dinitrophenyl)-L-2,3-diaminopropionyl and TFA is trifluoroacetic acid (Merck Millipore, Burlington, MA,), was added at a final concentration of 15 *μ*M to the reaction, and fluorescence was monitored using a Synergy 2 plate reader with 340/30 excitation and 400/30 emission filters (BioTek, Winooski, VT) at 37° C. Reactions were followed spectroscopically for 60 min, and initial rates were determined from the linear portion of the increase in fluorescence signal caused by cleavage of the fluorescent substrate. Data were globally fitted by multiple regression to Morrison's tight binding inhibition equation (see Eq. 1) using Prism version 5.00 for Windows (GraphPad, La Jolla, CA). *K_i_* values were calculated by plotting the initial velocities against different concentrations of the inhibitors. Reported inhibition constants represent average values obtained from three independent experiments. Calculations were performed using *K_m_* values of 9.5 ± 2.5 μM for MMP14_CAT_ and 5.5 ± 1.8 μM for MMP9_CAT_, as determined from at least three Michaelis–Menten kinetic experiments performed in our laboratory.

(Eq.1)

$$\frac{V_{i}}{V_{0}}=1-\frac{(\left[E\right]+\left[I\right]+K_{i}^{app})-\sqrt{{\left(\left[E\right]+\left[I\right]+K_{i}^{app}\right)}^{2}-4\left[E\right]\left[I\right]}}{2\left[E\right]}$$

where V_i_ is enzyme velocity in the presence of inhibitor, V_0_ is enzyme velocity in the absence of inhibitor, E is enzyme concentration, I is inhibitor concentration, S is substrate concentration, K_m_ is the Michaelis-Menten constant; and *K_i_*^app^ is the apparent inhibition constant, given by:

$$K_{i}^{app}=\;K_{i}(1+\frac{\left[S\right]}{K_{m}}$$

### Cell culture

MCF-7 human breast cancer cell line (a generous gift from Dan Levy, Ben-Gurion University) and U-87MG cells (American Type Culture Collection, Manassas, VA) were maintained in Dulbecco's modified Eagle's medium (DMEM; Biological Industries Beit-Haemek, Israel) supplemented with 10% fetal bovine serum (Thermo Fisher), 1% l-glutamine (Biological Industries) and 1% penicillin/streptomycin (Biological Industries).

### Stable cell line generation

For generating stable MCF-7 cells expressing either MMP14 or MMP9, MCF-7 cells were seeded at a density of 10^5^ and incubated for 24 h. The cells were then transfected with either plasmid pCMV MMP14 or plasmid pCMV MMP9 (Sino Biological, Beijing China) using Lipofectamin 3000 (Thermo Fisher) according to the manufacturer's instructions. Forty-eight h post-transfection, the cells were treated with 150 μg/ml of hygromycin (Thermo Fisher) followed by an incubation of 4 weeks. For assessing the expression of MMP14, MCF7 and MCF7-MMP14 cells were incubated for 1 h at room temperature with monoclonal anti-MMP14 rabbit antibodies (Abgent, San Diego, CA) following washing. The cells were then incubated for 30 min with secondary PE-conjugated goat-anti-rabbit antibodies (Abcam) and analyzed on an Accuri C6 flow cytometer (BD Biosciences).

### Gelatinase zymography assay

To test inhibition of endogenous MMP2 activation, a gelatin zymography assay was performed. U87MG cells (2×10^4^) were grown for 48 h in serum-free DMEM in the presence or absence of 100 nM of the protein inhibitors. The supernatants were collected and resolved by 7% SDS-PAGE in the presence of 1% embedded gelatin (Sigma). The gel was rinsed for 1 h with gentle agitation in 2.5% Triton X-100 (Thermo Fisher) at room temperature and incubated overnight in 50 mM Tris-HCl, pH 7.5, 10 mM CaCl_2_ and 100 mM NaCl at 37° C.

To examine inhibition of MMP9 activity by the N-TIMP2-based selective variants, 2 × 10^4^ MCF-7-MMP9 cells were grown in serum-free DMEM for 24 h. The supernatants were collected and resolved by 8% SDS-PAGE in the presence of 1% embedded gelatin. Afterwards, the gels were rinsed for 1 h with gentle agitation in 2.5% Triton X-100 at room temperature and incubated overnight with 100 nM of the N-TIMP2 inhibitors in 50 mM Tris-HCl, pH 7.5, 10 mM CaCl_2_ and 100 mM NaCl at 37° C. After incubation, the gels were stained with Simply-Blue SafeStain (Thermo Fisher), and gelatinolytic activity was visualized as clear bands. The signals obtained were quantified using ImageJ software. Validation of MMP9 expression in MCF-7 and MCF-7-MMP9 cells was performed by the same method.

### Trans-well migration assays

Trans-well migration assays were performed using ThinCert 24-well inserts (Greiner Bio-One, Kremsmünster, Austria). The bottom part of each insert was filled with DMEM supplemented with 10% fetal bovine serum. The upper compartment was filled with 200 *μ*l of MCF7, MCF7-MMP14 or MCF7-MMP9 cells (2 × 10^4^) in the presence or absence of the inhibitors at concentrations of 250, 100, 50 and 10 nM. The cells were incubated for 24 h at 37° C. Thereafter, cells that had migrated were fixed, stained using a Dipp Kwik Differential Stain Kit (American Mastertech Scientific, Lodi, CA) and counted using an EVOS FL Cell Imaging System (Thermo Fisher) at 20× magnification. The experiment was performed in triplicate, with 10 fields being counted for each sample.

### Data analysis and statistics

All experiments were performed in triplicate. The data was analyzed using GraphPad Prism version 5.00 for Windows. Statistical significance was determined by column statistics and *t* test analysis. A *P* value < 0.05 was considered statistically significant.

## SUPPLEMENTARY MATERIALS AND FIGURES



## Abbreviations

FACS: fluorescence-activated cell sorting
FITC: fluorescein isothiocyanate
MFI: mean fluorescence intensity
MMP: matrix metalloprotease
PE: phycoerythrin
PPI: protein–protein interaction
RU: response unit
SDS-PAGE: sodium dodecyl sulfate polyacrylamide gel electrophoresis
SEC: size-exclusion chromatography
TIMP: tissue inhibitor of matrix metalloproteases
YSD: yeast surface display.
