# Supplementary material for: Combinatorial engineering of N-TIMP2 variants that selectively inhibit MMP9 and MMP14 function in the cell
Source: Oncotarget. 2018 Aug 10;9(62):32036–53. doi: 10.18632/oncotarget.25885 (PMC6112833; doi:10.18632/oncotarget.25885)
Supplement: Supplementary file 1 [file oncotarget-09-32036-s001.pdf]

Combinatorial engineering of N-TIMP2 variants that selectively inhibit MMP9 and MMP14 function in the cell

SUPPLEMENTARY MATERIALS

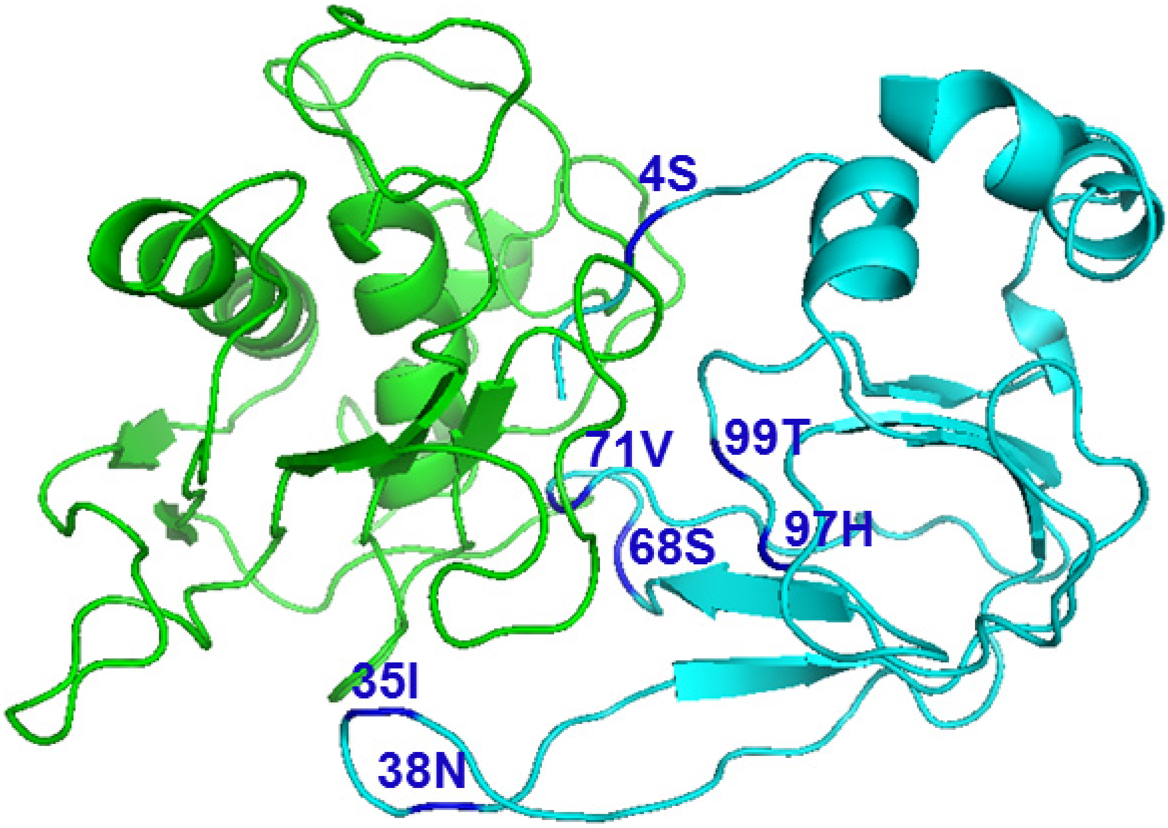

Supplementary Figure 1: Library design. Structure of N-TIMP2 (shown in cyan) in complex with $MMP14_{CAT}$ (shown in green) [adapted from PDB ID: 1BUV [58]], the positions chosen for full randomization are shown in blue.

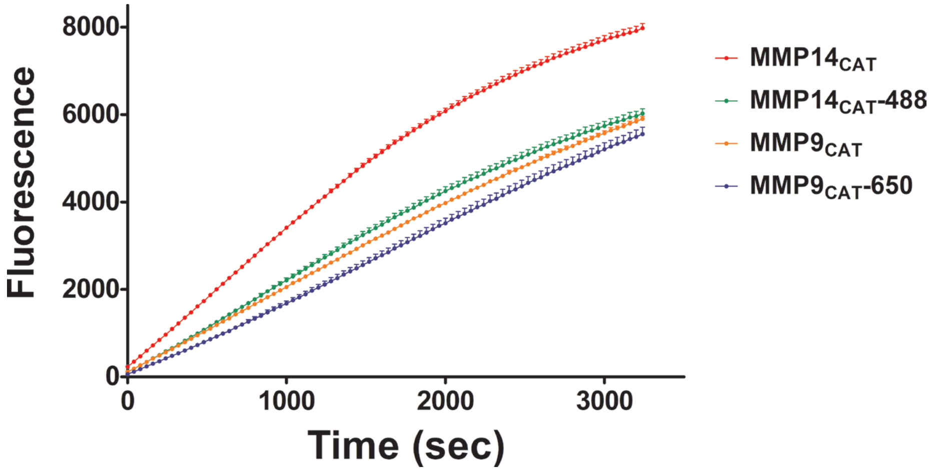

Supplementary Figure 2: MMP activity assay. Cleavage of the fluorogenic substrate Mca-Pro-Leu-Gly-Leu-Dpa-Ala-Arg-NH_2·TFA (340/30 excitation and 400/30 emission filters) at 37° C by $MMP14_{CAT}$ (red), DyLight-488 conjugated $MMP14_{CAT}$ (green), $MMP9_{CAT}$ (orange) and DyLight-650 conjugated $MMP9_{CAT}$.

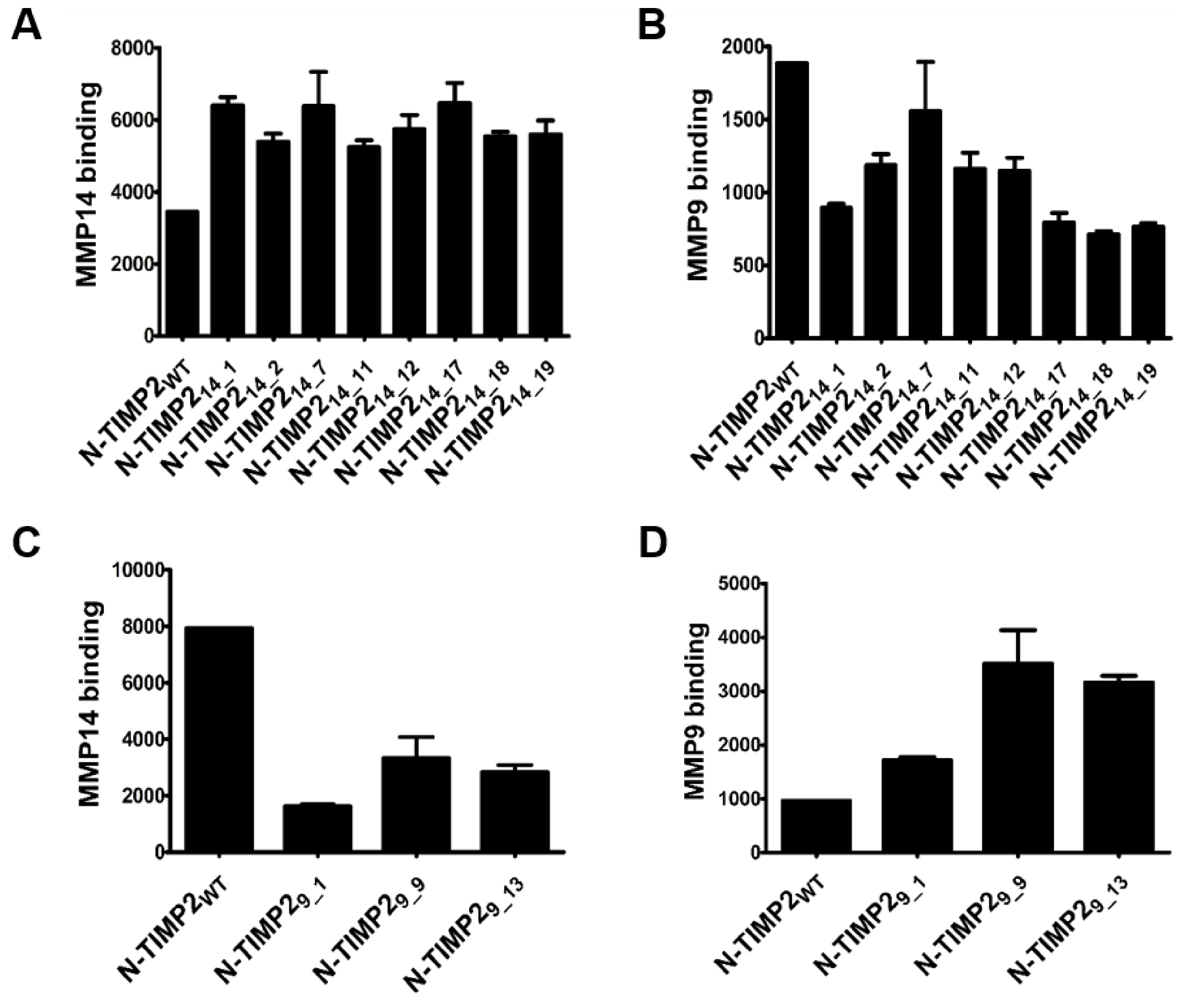

Supplementary Figure 3: YSD binding of individual clones. (**A**) YSD binding signal of MMP14-selective clones towards MMP14, as identified after the fourth round of sorting and analyzed at 100 nM of MMP14$_{CAT}$-488. (**B**) YSD binding signal towards MMP9 of MMP14-selective clones analyzed at 100 nM of MMP9$_{CAT}$-650. (**C**) YSD binding signal towards MMP14CAT of MMP9-binding variants identified after the fourth round of sorting analyzed at 1000 nM MMP14$_{CAT}$-488. (**D**) YSD binding signal towards MMP9 of MMP9-binding variants identified after the fourth round of sorting analyzed at 10 nM MMP9$_{CAT}$-650.

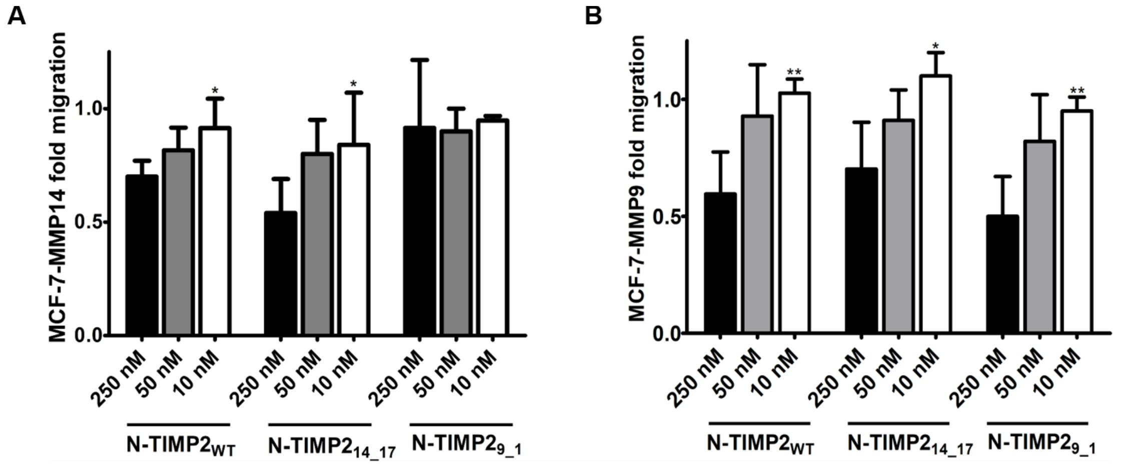

Supplementary Figure 4: Dose-dependent selective inhibition of MMP14- and MMP9-induced migration. (**A**) Calculated fold of migration, relative to the untreated control of MCF-7-MMP14 cells incubated with 250, 50 and 10 nM of the N-TIMP2$_{WT}$, N-TIMP2$_{14_17}$ and N-TIMP2$_{9_13}$ inhibitors. The cells were counted using ImageJ software and normalized to counts of untreated cells. (**B**) Calculated fold of migration of MCF-7-MMP9 cells incubated with 250, 50 and 10 nM of the N-TIMP2$_{WT}$, N-TIMP2$_{14_17}$ and N-TIMP2$_{9_13}$ inhibitors. The cells were counted as in panel (A). $^{*}P < 0.05$ and $^{**}P < 0.01$, as determined in *t* tests comparing the fold migration at concentration of 250 nM inhibitor and 10nM inhibitor for each variant.
